# Supplementary material for: Impact of the gut microbiota on the m6A epitranscriptome of mouse cecum and liver
Source: Nat Commun. 2020 Mar 12;11:1344. doi: 10.1038/s41467-020-15126-x (PMC7067863; doi:10.1038/s41467-020-15126-x)
Supplement: Supplementary file 1 — Supplementary Information [file 41467_2020_15126_MOESM1_ESM.pdf]

Supplementary Information for

Impact of the gut microbiota on the m^{6}A epitranscriptome of mouse cecum and liver

Jabs et al.

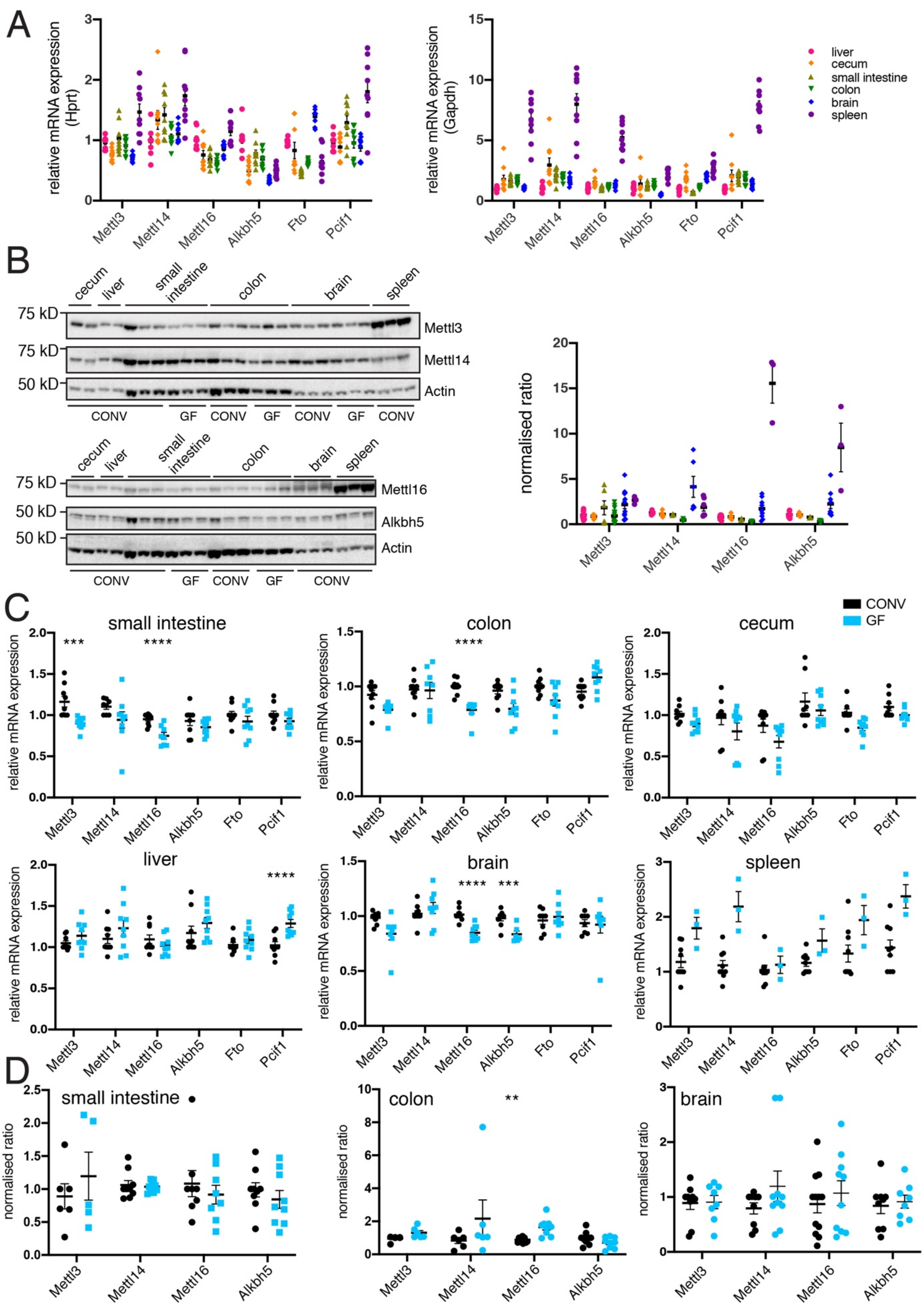
A
relative mRNA expression (Hprt)
relative mRNA expression (Gapdh)
Mettl3
Mettl14
Mettl16
Alkbh5
Fto
Pcif1
liver
cecum
small intestine
colon
brain
spleen
B
cecum
liver
small intestine
colon
brain
spleen
75 kD
50 kD
Mettl3
Mettl14
Actin
CONV
GF
Mettl16
Alkbh5
normalised ratio
C
CONV
GF
small intestine
colon
cecum
liver
brain
spleen
relative mRNA expression
D
normalised ratio

Supplementary Figure 1
Tissue expression of different methyltransferases and demethylases

A, relative mRNA expression of different methyltransferases and demethylases in small intestine (olive triangles), colon (green triangles), cecum (orange diamond), liver (pink circle), brain (blue diamonds) and spleen (violet circles) by qRT PCR. Two different housekeeping genes (Hprt, Gapdh) were used to compare expression between different tissues. *n*=9 from 3 different mice, except small intestine Mettl14 and cecum Fto: *n*=8; three independent qRT PCR experiments were performed. **B,** Tissue protein expression of Mettl3, Mettl14, Mettl16 and Alkbh5. For quantification, protein of interest/ actin ratios were normalised to their respective expression in cecum or liver. Mettl3: cecum *n*=10; liver *n*=13; small intestine *n*=6; colon *n*=12; brain *n*=12; spleen *n*=3; Mettl14: cecum *n*=8; liver *n*=7; small intestine *n*=6; colon *n*=3; brain *n*=6; spleen *n*=6; Mettl16 cecum *n*=6; liver *n*=9 ; small intestine *n*=3; colon *n*=3; brain *n*=9; spleen *n*=3; Alkbh5: cecum *n*=9; liver *n*=8; small intestine *n*=3; colon *n*=6 ; brain *n*=9; spleen *n*=3**,** all from 3 different mice; **C**, relative mRNA expression of different methyltransferases and demethylases in small intestine, colon, cecum, liver brain and spleen by qRT PCR of CONV (black) and GF (cyan) mice. Cecum, colon, liver, brain, spleen (CONV) n=9; small intestine (CONV), all *n*=9 except Mettl14: *n*=8; colon: Mettl3, Mettl14, Mettl16 *n*=8; brain: Mettl3, Fto, Pcif1 *n*=8; Alkbh5 *n*=7; all from 3 different mice; spleen (GF): *n*=3 (1 mouse); in all cases, three independent qRT PCR experiments were performed. Multiple unpaired t-tests were performed for statistical analysis of differential expression between CONV and GF (except for the spleen); Holmak-Sidiak-adjusted *p*-values: * <0.05; ** <0.01; *** <0.05; *** <0.0001; small intestine: *p*= 0.00830 (Mettl3); 0.46435 (Mettl14); 0.00323 (Mettl16); 0.46435 (Alkbh5); 0.46435 (Fto); 0.46435 (Pcif); cecum *p*= 0.00167 (Mettl3); 0.41020 (Mettl14); 0.26902 (Mettl16); 0.41020 (Alkbh5); 0.04490 (Fto); 0.24889 (Pcif); colon: *p*= 0.05270 (Mettl3); 0.91961 (Mettl14); 0.00037 (Mettl16); 0.05270 (Alkbh5); 0.06431 (Fto); 0.05270 (Pcif); liver: *p*= 0.604 (Mettl3); 0.691 (Mettl14); 0.691 (Mettl16); 0.691 (Alkbh5); 0.691 (Fto); 0.006 (Pcif); brain *p*= 0.02930 (Mettl3); 0.37915 (Mettl14); 0.00015 (Mettl16); 0.00409 (Alkbh5); 0.47148 (Fto); 0.58669 (Pcif); **D**, protein expression of Mettl3, Mettl14, Mettl16 and Alkbh5 in CONV and GF mice in indicated tissues. Two to four independent Western Blots were performed; small intestine: Mettl3: CONV *n*=6; GF *n*=5; Mettl14: CONV *n*=9; GF *n*=9; Mettl16: CONV *n*=9; GF *n*=9; Alkbh5: CONV *n*=9; GF *n*=8; colon: Mettl3: CONV *n*=6; GF *n*=6; Mettl14: CONV *n*=6; GF *n*=5; Mettl16: CONV *n*=9; GF *n*=9; Alkbh5: CONV *n*=9; GF *n*=9; brain: Mettl3: CONV *n*=9; GF *n*=8; Mettl14: CONV *n*=9; GF *n*=10; Mettl16: CONV *n*=12; GF *n*=10; Alkbh5: CONV *n*=9; GF *n*=8; 3 different mice; Multiple unpaired t-tests were performed for statistical analysis of differential expression between CONV and GF; Holmak-Sidiak-adjusted *p*-value: ** <0.01. *p*-value small intestine 0.453 (Mettl3); 0.754 (Mettl14); 0.506 (Mettl16); 0.412 (Alkbh5); *p*-value colon 0.041 (Mettl3); 0.272 (Mettl14); 0.00057 (Mettl16); 0.151 (Alkbh5); *p*-value brain 0.919 (Mettl3); 0.213 (Mettl14); 0.474 (Mettl16); 0.683 (Alkbh5); Original data and details for statistical analysis are given in the source data file. Data are presented as mean +/- SEM throughout the figure.

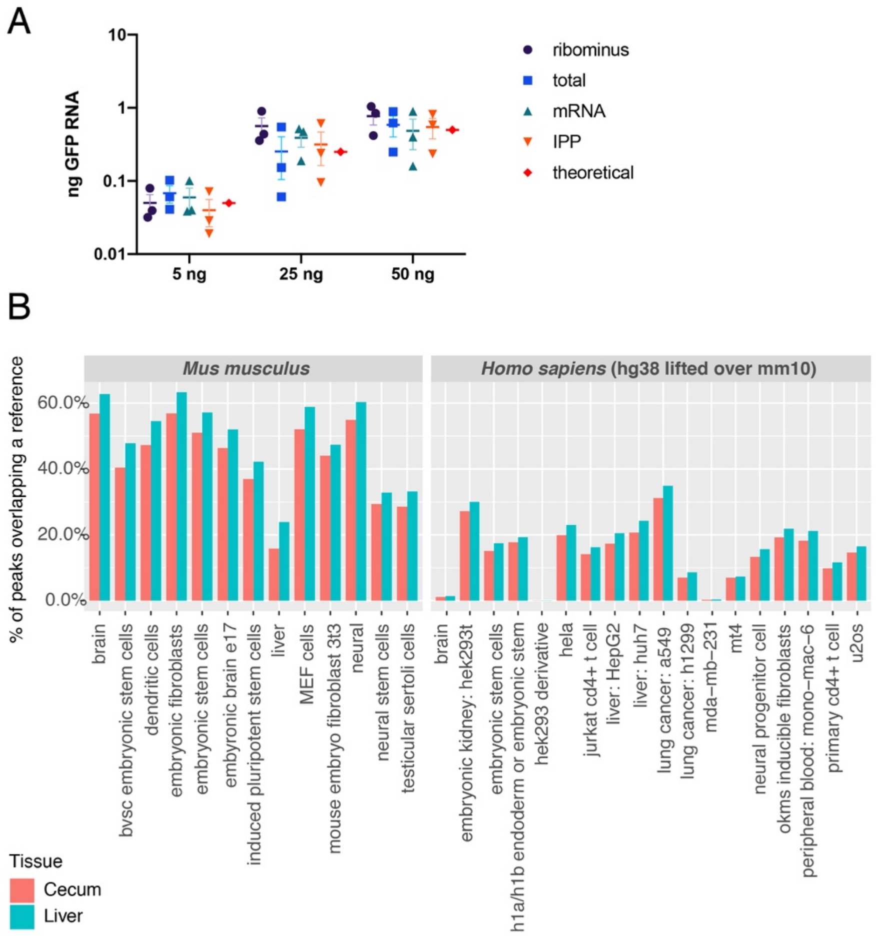

Supplementary Figure 2

m^{6}A differential methylation analysis, overlap with published peaks

A, methylated RNA-IP of 5, 25, or 50 ng of *in vitro* transcribed m^{6}A-containing GFP-transcripts from samples of 5 µg ribodepleted (black circles), 200 µg total (blue squares) and 5 µg mRNA (green triangles), or immunoprecipitation buffer (IPP ; orange triangles) quantified by qRT PCR. Theoretical yield is indicated (theoretical; red diamond). Data are presented as mean +/- SEM; 2way ANOVA for multiple comparisons was performed; details and data are given in the source data file; **B**, overlap all m^{6}A peaks we detected in our 64 datasets from cecum and 33 datasets from the liver with datasets from murine (left panel) and human (right panel) tissues and cells published in the MeT-DB v2.0 database[1] were determined. Our detected peaks may overlap with several published peaks. To compare our peaks from murine tissue with corresponding human peaks, the *Homo sapiens* hg38 genome was lifted over the *Mus musculus* mm10 genome.

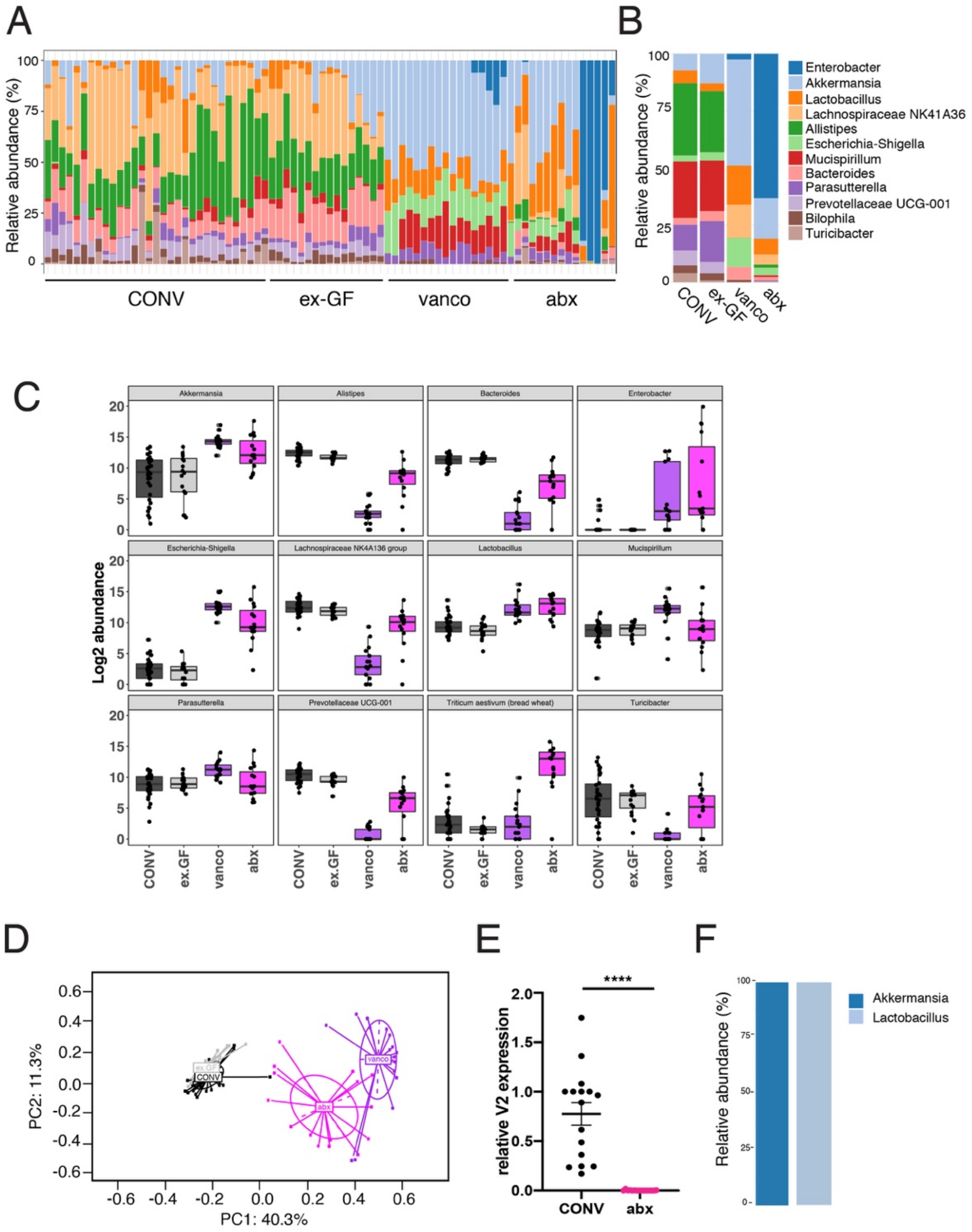

A
B
Relative abundance (%)
CONV
ex-GF
vanco
abx
Enterobacter
Akkermansia
Lactobacillus
Lachnospiraceae NK41A36
Allistipes
Escherichia-Shigella
Mucispirillum
Bacteroides
Parasutterella
Prevotellaceae UCG-001
Bilophila
Turicibacter
C
Log2 abundance
Akkermansia
Alistipes
Bacteroides
Enterobacter
Escherichia-Shigella
Lachnospiraceae NK4A136 group
Lactobacillus
Mucispirillum
Parasutterella
Prevotellaceae UCG-001
Triticum aestivum (bread wheat)
Turicibacter
D
PC1: 40.3%
PC2: 11.3%
E
relative V2 expression
****
F
Akkermansia
Lactobacillus

Supplementary Figure 3
Gut microbiota composition
A, B, most abundant genera in cecal content of CONV (*n*=30); ex-GF mice colonized with CONV flora for 4 weeks (*n*=16; 3 independent experiments), vancomycin/amphotericin B-treated mice (*n*= 12; 4 independent experiments) and abx mice that had been treated with vancomycin, metronidazol, neomycin, ampicillin and amphotericin B every 12h for 21 days (*n*=10, 4 independent experiments); **A**, individual samples; **B**, mean; **C,** individual boxplots for most abundant genera in CONV, ex-GF, vanco and abx mice; for n see **A**) The lower, central and upper hinges correspond to the first, second (median) and third quartiles. The upper and lower whiskers, respectively, correspond to the higher and lower values at the 1.5 interquartile range from the hinge (where the interquartile range is the distance between the first and third quartiles); **D**, Principal coordinates analysis of samples from **A; E**, quantitative PCR for V2 region of cecal content from antibiotics- (abx, *n*=16) and vehicle treated (*n*=16) mice from four independent experiments. Relative expression compared to mouse mpI genomic region. **** *p*-value<0.001 (*p*=0.000034) unpaired two-tailed t-test. Details for statistical analysis and original data are given in the source data file; **F**, 16S analysis from cecal content of mice monoassociated with *A.muciniphila*-, and *L.plantarum* showing successful monocolonisation (*A.muciniphila*: *n*=12; *L.plantarum*: *n*=8*).*

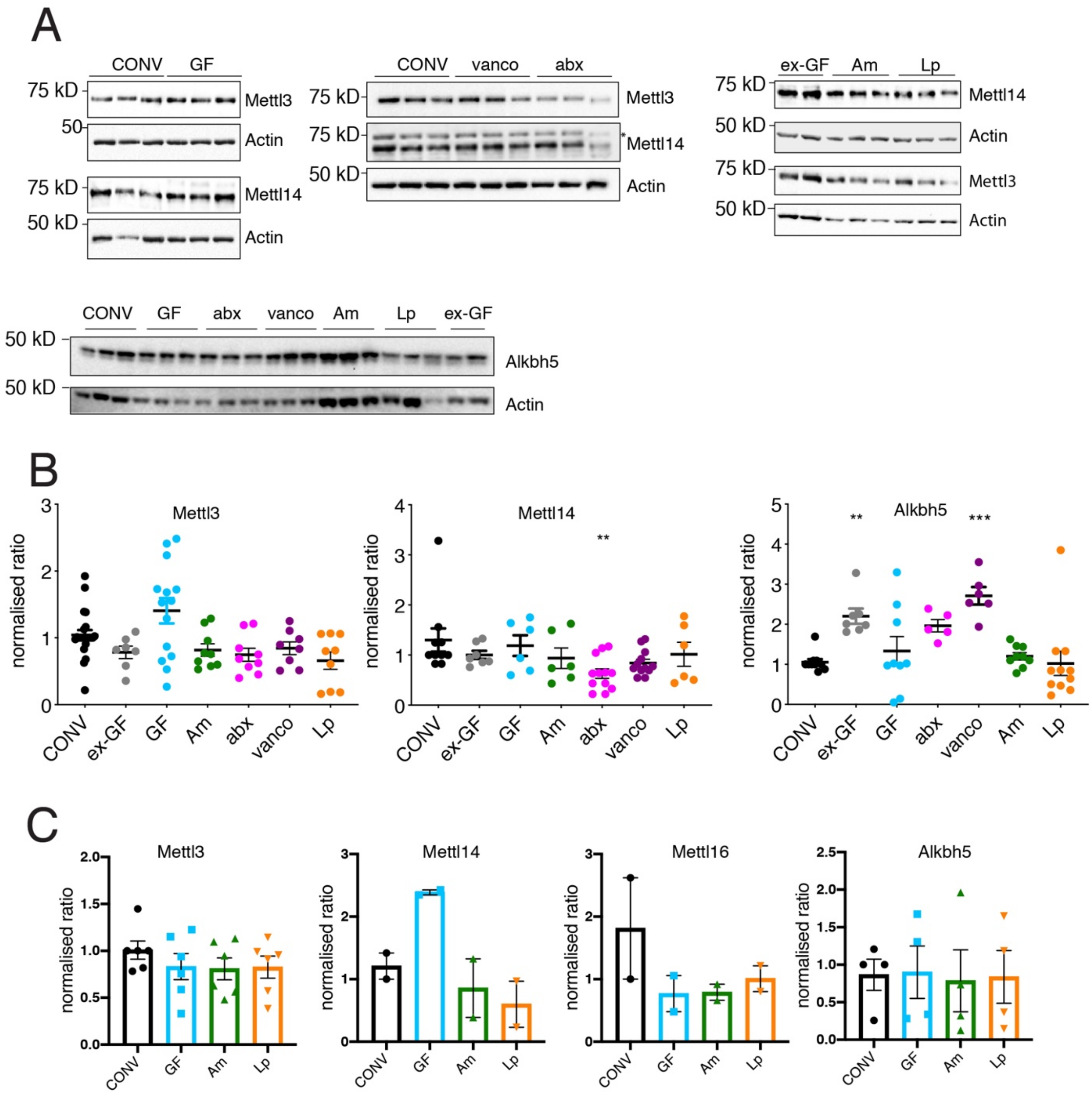

Supplementary Figure 4

Tissue expression of methyltransferases Mettl3, Mettl14 Mettl16, and Alkbh5a in all mouse models in cecum and liver

A, Western Blot analysis of Mettl3, Mettl14 and Alkbh5 expression from ceca of CONV, GF, ex-GF, abx, vanco, Am, and Lp mice. Actin served as loading control. The membrane and thus the actin loading control for the blot displaying samples from ex-GF, Am, and Lp mice is identical to western blot shown in Figure 4A. **B**, quantification of Western Blots shown in **A**; protein of interest/ actin ratio was normalised to ratio in CONV mice. Mettl3: CONV *n*=23, ex-GF *n*=7; GF *n*=14; abx *n*= 9; vanco *n*=8; Am *n*=9; Lp *n*=9; Mettl14: CONV *n*=12, ex-GF *n*=7; GF *n*=6; abx *n*= 9; vanco *n*=9; Am *n*=6; Lp *n*=6; Alkbh5: CONV *n*=10, ex-GF *n*=5; GF *n*=9; abx *n*= 5; vanco *n*=6; Am *n*=9; Lp *n*=11; Ordinary one-way ANOVA was performed. *** <0.0001, ** *p*-value <0.005 (Holm-Sidak's multiple comparisons test) ; Mettl3 *p*-values (all compared to CONV): ex-GF: 0.3897; GF: 0.0747; abx : 0.3897; vanco : 0.2652; Am : 0.3897; Lp : 0.1058; Mettl14

p-values (all compared to CONV): ex-GF: 0.4914 ; GF: 0.6463 ; abx : 0.0089; vanco : 0.1249 ; Am : 0.4487 ; Lp : 0.491; Alkbh5 *p*-values (all compared to CONV): ex-GF: 0.0069; GF: 0.7603; abx : 0.0757; vanco : 0.0001; Am : 0.8758; Lp : 0.8758; **C**, quantification of Western Blots for CONV, GF, Am and Lp liver; protein of interest/ actin ratio was normalised to ratio in CONV mice. Mettl3: CONV n=*6*, GF *n*=6; Am *n*=6; Lp *n*=6; Mettl14: CONV *n*=2, GF *n*=2; Am *n*=2; Lp *n*=2; Mettl16: CONV *n*=2, GF *n*=2; Am *n*=2; Lp *n*=2; Alkbh5: CONV *n*=4, GF *n*=4; Am *n*=4; Lp *n*=4; due to low number of replicates, statistical analysis was only performed for Mettl3; details and all original blots are given in the source data file.

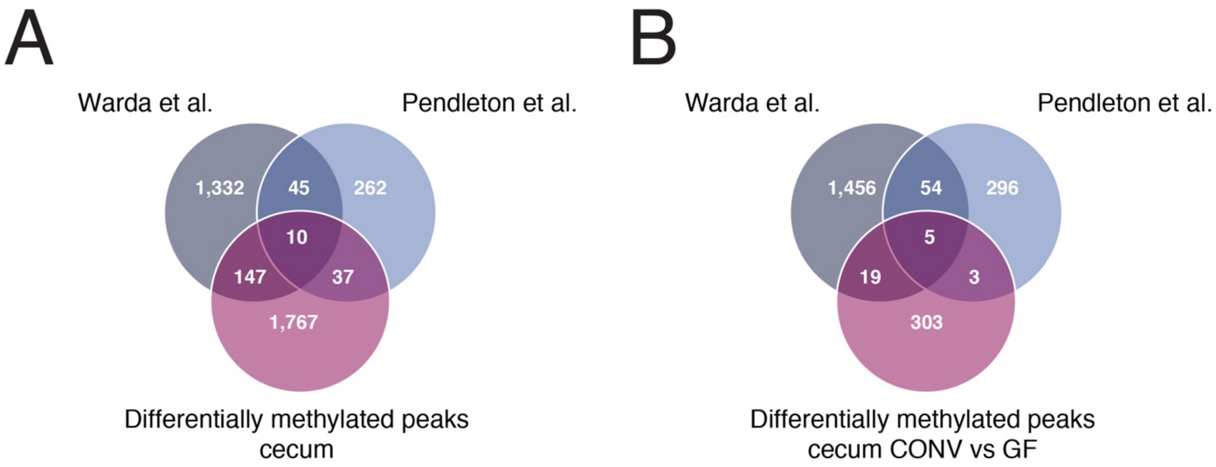

Supplementary Figure 5
Overlap of differentially methylated peaks with METTL16 targets

A, Overlap of putative METTL16 targets described in Warda et al.[2] and Pendleton et al.[3] with differentially methylated peaks in cecum across all conditions, or **B**, in CONV vs GF mice.

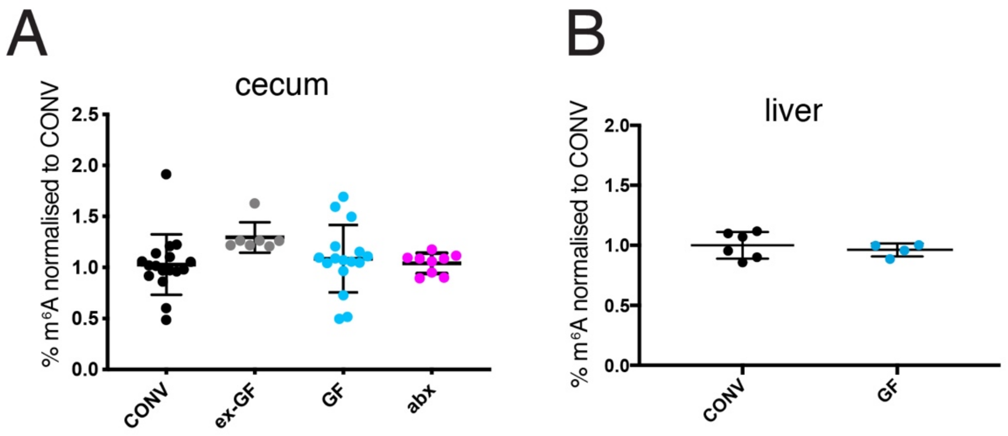

Supplementary Figure 6
Quantification of selected mRNA modifications by liquid chromatography coupled to high resolution mass spectromtry (LC-HRMS)

LC-HRMS of ribodepleted RNAs from **A,** cecum (CONV *n*=17, GF *n*=16, ex-GF *n*=7; abx *n*=9) and **B,** liver (CONV *n*=6; GF *n*=4); due to variability between MS analyses, samples for each batch (three in total) were normalised to the mean of the CONV values. Error bars depict SEM. Values are given in the source data file.

Supplementary references

1 Liu, H. *et al.* MeT-DB V2.0: elucidating context-specific functions of N6-methyl-adenosine methyltranscriptome. *Nucleic Acids Research* **46**, D281-D287(2018).
2 Warda, A. S. *et al.* Human METTL16 is a N⁶-methyladenosine (m⁶A) methyltransferase that targets pre-mRNAs and various non-coding RNAs. *EMBO reports* **18**, 2004-2014 (2017).
3 Pendleton, K. E. *et al.* The U6 snRNA m6A Methyltransferase METTL16 Regulates SAM Synthetase Intron Retention. *Cell* **169**, 824-835.e814 (2017).
4 Chen, E. Y. *et al.* Enrichr: interactive and collaborative HTML5 gene list enrichment analysis tool. *BMC Bioinformatics* **14**, 128 (2013).
5 Kuleshov, M. V. *et al.* Enrichr: a comprehensive gene set enrichment analysis web server 2016 update. *Nucleic Acids Research* **44**, W90-W97(2016).
