# Supplementary material for: Impact of the gut microbiota on the m6A epitranscriptome of mouse cecum and liver
Source: Nat Commun. 2020 Mar 12;11:1344. doi: 10.1038/s41467-020-15126-x (PMC7067863; doi:10.1038/s41467-020-15126-x)
Supplement: Supplementary file 3 — Description of Additional Supplementary Files [file 41467_2020_15126_MOESM3_ESM.pdf]

Description of Additional Supplementary Files

File Name: Supplementary Data 1
Description: **Quality check RNA sequencing data cecum and liver.** This table contains the mapping statistics obtained with STAR (for the whole transcriptome) and bowtie2 (for ribosomal RNAs), the sequencing depth, and the quality statistics of the libraries obtained with FastQC and RSeQC

File Name: Supplementary Data 2
Description: **Data table differential methylation and expression in cecum and liver.** This table contains the raw read counts for methylation peaks in IP and input samples (sheets "cecum raw counts" and "liver raw counts") and the log fold changes and the false discovery rate of the differential analysis (sheets "cecum differential" and "liver differential") performed by limma-voom for methylation peaks in IP samples and for genes in input samples.

File Name: Supplementary Data 3
Description: **Differential transcript usage cecum and liver.**This table gives an overview of the isoforms showing evidence of differential usage that are overlapping with differential m^6A/m peaks.The first sheet gives for each comparison and each tissue the number of: genes found differentially expressed (column *nbDEgenes*), peaks found differentially methylated (column *DMpeaks*), and genes showing evidence of differential transcript usage (column *nbDTUgenes*), and the number of genes present in the intersection of these different categories. The following sheet gives, for each comparison within each tissue, a description of the peaks overlapping genes that show evidence of differential isoform usage. The colum *peaks* gives the list of peak IDs, the columns *geneID*, *gene_name,* and *txID* give the IDs of the genes and transcript isoforms they overlap with, the column *DTU_gene_qval* gives the q-value output by DEXSeq for each gene, the column *DTU_tx_OFDR* gives the overall false discovery rate output by stageR for each isoform, the two columns *DTU_mean_IF* gives the average isoform fraction within each condition, the *DGE_gene_FDR* column gives the FDR output by voom for evidence of differential expression, the *DGE_gene_status* gives the information of whether the gene was considered as differentially expressed (only genes with an absolute log fold change larger than 1 were considered differentially expressed) ; the following columns give additional annotations about genes and peaks.

File Name: Supplementary Data 4
Description: **Pathway analysis of differentially methylated transcripts in cecum.** Pathway analysis of molecular and cellular function of cecal transcripts differentially methylated between CONV and GF (CONV vs GF), GF and Lp (Lp vs GF), and GF and Am (Am vs GF) mice was performed using Ingenuity pathway analysis (IPA). Category of the enriched pathway, Benjamini-Hochberg-corrected *p*-values (B-H p-value), and differentially methylated transcripts (molecules) are displayed. For CONV vs GF, we additionally performed pathway analysis for molecular and cellular function and diseases and disorders. Corresponding graphs are displayed in Figure 3A, F and G.

File Name: Supplementary Data 5
Description: **Pathway analysis of differentially methylated transcripts in liver.** Pathway analysis of molecular and cellular function of liver transcripts differentially methylated between CONV and GF (CONV vs GF), CONV and Am (CONV vs Am), CONV and Lp (CONV vs Lp) and GF and Lp (GF vs Lp) mice was performed using Enrichr[4,5]. Category of the enriched pathway, P-values, adjusted *p*-values, odds ratio, combined score and affected transcripts (genes) are displayed.

File Name: Supplementary Data 6
Description: **Normalized count tables on genus and OTU level.** Normalized counts from 16S sequencing are displayed for the individual samples from CONV (*n*=16), vanco (n=12) and abx (*n*=10) cecal content.
