# Supplementary material for: Impact of the gut microbiota on the m6A epitranscriptome of mouse cecum and liver
Source: Nat Commun. 2020 Mar 12;11:1344. doi: 10.1038/s41467-020-15126-x (PMC7067863; doi:10.1038/s41467-020-15126-x)
Supplement: Supplementary file 10 — Reporting Summary [file 41467_2020_15126_MOESM10_ESM.pdf]

nature research

Corresponding author(s): Sabrina Jabs, Pascale Cossart

Last updated by author(s): 2020/02/14

Reporting Summary

Nature Research wishes to improve the reproducibility of the work that we publish. This form provides structure for consistency and transparency in reporting. For further information on Nature Research policies, see Authors & Referees and the Editorial Policy Checklist .

Statistics

For all statistical analyses, confirm that the following items are present in the figure legend, table legend, main text, or Methods section.

n/a	Confirmed	
☐	☒	The exact sample size (*n*) for each experimental group/condition, given as a discrete number and unit of measurement
☐	☒	A statement on whether measurements were taken from distinct samples or whether the same sample was measured repeatedly
☐	☒	The statistical test(s) used AND whether they are one- or two-sided<br>*Only common tests should be described solely by name; describe more complex techniques in the Methods section.*
☐	☒	A description of all covariates tested
☐	☒	A description of any assumptions or corrections, such as tests of normality and adjustment for multiple comparisons
☐	☒	A full description of the statistical parameters including central tendency (e.g. means) or other basic estimates (e.g. regression coefficient) AND variation (e.g. standard deviation) or associated estimates of uncertainty (e.g. confidence intervals)
☐	☒	For null hypothesis testing, the test statistic (e.g. *F*, *t*, *r*) with confidence intervals, effect sizes, degrees of freedom and *P* value noted<br>*Give P values as exact values whenever suitable.*
☒	☐	For Bayesian analysis, information on the choice of priors and Markov chain Monte Carlo settings
☒	☐	For hierarchical and complex designs, identification of the appropriate level for tests and full reporting of outcomes
☒	☐	Estimates of effect sizes (e.g. Cohen's *d*, Pearson's *r*), indicating how they were calculated

Our web collection on statistics for biologists contains articles on many of the points above.

Software and code

Policy information about availability of computer code

Data collection	Andromeda with the MaxQuant software version 1.5.3.8, STAR mapper 2.5.0a, SAMtools 0.1.19 , Alientrimmer (v0.4.0), VSEARCH (v2.3.4),FastQC 0.10.1 ,MultiQC 0.7, HTSeq 0.9, BEDTools 2.17.0, MACS2.
Data analysis	All the scripts used for m6A peak detection and methylation peak analysis have been deposited on Institut Pasteur GitLab: https://gitlab.pasteur.fr/hub/MeRIPSeq. The bioinformatic workflow work 16S sequencing analysis is available at https://github.com/aghozlane/masque; furthermore, we used GraphPad Prism 8 for macOS (Version 8.31);Ingenuity Pathway analysis; Enrichr http://amp.pharm.mssm.edu/Enrichr/; Integrative genomics viewer IGV2.4.14; GUITAR http://bioconductor.org/packages/release/bioc/html/Guitar.html; limma https://bioconductor.org/packages/release/bioc/html/limma.html; voom https://bioconductor.org/packages/release/bioc/html/limma.html; MEME, DREME.

For manuscripts utilizing custom algorithms or software that are central to the research but not yet described in published literature, software must be made available to editors/reviewers. We strongly encourage code deposition in a community repository (e.g. GitHub). See the Nature Research guidelines for submitting code & software for further information.

Data

Policy information about availability of data

All manuscripts must include a data availability statement. This statement should provide the following information, where applicable:
- Accession codes, unique identifiers, or web links for publicly available datasets
- A list of figures that have associated raw data
- A description of any restrictions on data availability

MeRIP-Seq and RNA-seq data have been deposited in the ArrayExpress database at EMBL-EBI (www.ebi.ac.uk/arrayexpress) under accession number E-MTAB-6560. 16S rRNA sequencing data have been deposited in the European Nucleotide Archive database at EMBL-EBI (https://www.ebi.ac.uk/ena) under accession number PRJEB25147. 16S data are also available in SHAMAN (shaman.pasteur.fr; key ff9551570bf15) or Figshare https://doi.org/10.6084/m9.figshare.8321165.v5. The mass spectrometry proteomics data have been deposited to the ProteomeXchange Consortium via the PRIDE partner repository with the dataset identifier PXD016099. Proteomic data were searched against a uniprot database containing Mus musculus proteins (downloaded 03/2016; https://www.uniprot.org/proteomes/); public

databases used were MeT-DB v2.0 database http://180.208.58.19/metdb_v2/html/index.php; SILVA SSU (v128) https://www.arb-silva.de/documentation/release-128/; Gencode (human genome hg38 https://www.gencodegenes.org/human/releases.html; mouse mm10 genome and list of transcripts (Mus Musculus VM13) https://www.gencodegenes.org/mouse_releases/.); Mouse images in Figures 1 and 5 were obtained from https://www.wikihow.com/Draw-a-Mouse#/Image:Color-Step-7-5.jpg and are licensed in CreativeCommons by-nc-sa/2.5;
Source data for figures 4A, 4B, 4D, 4E, 4F,4G, and supplementary figures 1A,1B,1C, 1D, 2A, 3E, 4A, 4B, 4C, and 6 are given in the Source data file associated to this manuscript.

Field-specific reporting

Please select the one below that is the best fit for your research. If you are not sure, read the appropriate sections before making your selection.

- [x] Life sciences
- [ ] Behavioural & social sciences
- [ ] Ecological, evolutionary & environmental sciences

For a reference copy of the document with all sections, see nature.com/documents/nr-reporting-summary-flat.pdf

Life sciences study design

All studies must disclose on these points even when the disclosure is negative.

Sample size	Sample size estimates have been performed on previous experience and published results to obtain statistical significance and reproducibility, i.e. n>3 for MeRIP-seq, N=3 for qRT PCR, n>4 for 16S analysis; n>5 for proteomics analysis
Data exclusions	Only exclusions have been made in case of failed experimental procedures; e.g. MeRIPseq had very few reads and/or their IP samples had significantly more zero reads than the other samples (was the case for 1 vanco, 1 Am, 1 Lp sample); sequencing libraries with very low yield or bad profiles were not used for sequencing; in case of very unequal loading or uneven band shape/ signs of protein degradation, single samples were excluded from quantification of Western Blots (indicated in source data).
Replication	All experiments underlying main conclusions of this study have been successfully replicated multiiple times (3 for qRT, 3 for Western Blots unless indicated otherwise, 4 for antibiotics treatment and 16S analysis). MeRIPseq results were obtained from two independent datasets.
Randomization	Samples and organisms were randomly allocated to experimental groups. No specific randomization protocol has been used. Mice were age- and sex matched.
Blinding	No specific blinding was required.

Reporting for specific materials, systems and methods

We require information from authors about some types of materials, experimental systems and methods used in many studies. Here, indicate whether each material, system or method listed is relevant to your study. If you are not sure if a list item applies to your research, read the appropriate section before selecting a response.

Materials & experimental systems

n/a	Involved in the study	
[ ]	[x]	Antibodies
[x]	[ ]	Eukaryotic cell lines
[x]	[ ]	Palaeontology
[ ]	[x]	Animals and other organisms
[x]	[ ]	Human research participants
[x]	[ ]	Clinical data

Methods

n/a	Involved in the study	
[x]	[ ]	ChIP-seq
[x]	[ ]	Flow cytometry
[x]	[ ]	MRI-based neuroimaging

Antibodies

Antibodies used	Antibody used and dilutions were: rabbit anti Mettl3 (abcam ab195352), rabbit anti Mettl16 (abcam ab186012), rabbit anti Alkbh5 (abcam ab195377) and rabbit anti Mat2a (abcam ab154343): 1:1,000; mouse anti beta-actin (Sigma-Aldrich A1978): 1:2,000; rabbit anti Mettl14 (Sigma Aldrich HPA038002): 1:500.
Validation	Mettl3 and Alkbh5 antibodies were KO validated as stated by the provider.

Animals and other organisms

Policy information about studies involving animals; ARRIVE guidelines recommended for reporting animal research

Laboratory animals	C57BL/6J mice were used. Conventional mice were kept in specific pathogen- free conditions and all the mice used were age-matched and female. Mice were housed in 10h (dark)/ 14h (light) cycles.
Wild animals	No wild animals were used.
Field-collected samples	No field-collected samples were used.
Ethics oversight	All animal experiments were approved by the committee on animal experimentation of the Institut Pasteur and by the French Ministry of Agriculture.

Note that full information on the approval of the study protocol must also be provided in the manuscript.
